# Nursing faculty experiences with student incivility in South Korea: a qualitative study

**DOI:** 10.1186/s12909-022-03170-8

**Published:** 2022-02-18

**Authors:** Myung Sun Hyun, Hee Sun Kang, Jennie C. De Gagne, Jeonghwa Park

**Affiliations:** 1 Research Institute of Nursing Science, College of Nursing, Ajou University, 164, World Cup-ro, Yongtong-gu, Suwon-si, Gyeonggi-do 16499 Republic of Korea; 2 Red Cross College of Nursing, Chung-Ang University, 84 Heukseok-ro Dongjak-gu, Seoul, 06974 Republic of Korea; 3 Duke University School of Nursing, 307 Trent Drive, Durham, NC 27710 USA; 4 Department of Nursing, Pai Chai University, 155-40 Baejae-ro, Seo-Gu, Daejeon, 35345 Republic of Korea

**Keywords:** Incivility, Education, Faculty, Nursing, Qualitative research

## Abstract

**Background:**

Student academic incivility is a serious problem in nursing education because it negatively influences the learning process, wellbeing of faculty members, and faculty–student relationships. The aim of this study was to explore nursing faculty experiences related to student incivility in nursing education.

**Methods:**

We used an exploratory qualitative methodology and conducted in-depth interviews with 14 nursing faculty members working at 11 nursing universities in South Korea. A qualitative content analysis was carried out.

**Results:**

The six themes that emerged from the faculty experiences were nursing student indifference to teachers’ expectations; ignoring behavioral standards in school life; differing responses to uncivil student behavior; self-reflection as a nursing educator; negative impact on faculty in a nursing education context; and awareness of civility in a nursing education context.

**Conclusion:**

Dealing with student academic incivility in nursing education is one of the most challenging tasks for faculty members, and it should be managed to provide a comfortable learning environment. The present study delineated the uncivil behavior experienced by the faculty and their negative effects in the educational context and suggested alternatives to manage student incivility and foster a positive educational environment through the eyes of the faculty. The findings of this study suggest an evidence-based direction for fostering a culture of civility in nursing schools.

## Background

Along with the growing prevalence of general uncivil behavior, academic incivility is a major problem in nursing education [1]. Incivility in nursing education is defined as rude or disruptive behaviors that might cause psychological or physiological distress for the individuals involved [2]. Incivility in nursing education is considered an emergent and serious problem worldwide that may disrupt the learning environment and faculty-student relationships [3, 4].

Specifically, students’ incivility toward faculty has required increased attention [5]. Many scholars have emphasized the severity of this problem, as it potentially has multiple negative consequences [3, 6, 7]. Student incivility may negatively influence faculty members’ psychological states—such as self-esteem and peace of mind—by creating anxiety and depression—and cognitive functions such as the inability to think rationally; it could even lead to physical effects such as headaches, sleep disruption, and a weakened immune system [3, 7]. Student incivility might also adversely affect productivity among faculty members, lead them to question their careers as educators, undermine teacher–student relationships, and lower faculty recruitment and retention [5, 6]. Clark and Springer [2] noted that student incivility in nursing education that is ignored or not under control may lead to threatening clinical situations, adversely affecting patient safety, because students have direct contact with patients through clinical practicum. Therefore, academic incivility, specifically student incivility in nursing education, must be addressed to promote nursing education and ensure patient safety [5, 8].

Previous studies investigated faculty experiences with incivility [5, 6], and those in the area of nursing education were mostly conducted in Western countries [1]. However, behaviors defined as uncivil take various forms and have various meanings across cultures [1, 4], and they are based on subjectively perceived experience [3]. In South Korea, society is characterized by paternalism and hierarchy [9]. Korean people value harmony and bonds among members; they value emotional connections in human relationships. In addition, Korean people tend to value rank and yield to seniors. Korean faculty members, who can be regarded as senior, tend to relate to students in the same way elderly individuals in society relate to younger individuals [10]: they try to educate in the traditional way by protecting, supervising, and controlling students [11]. However, students of the millennial generation (also known as Generation Y or Gen Y, which refers to those born between 1981 and 1996) tend to follow their own values and assert themselves clearly, challenging their teachers’ authority [10]. Korean students tend to view education as a service provided by the school instead of knowledge acquisition for its own sake [10]. Being focused only on one’s own goals at the expense of other people, their egocentric behaviors are often perceived as selfish and uncivil [12]. This phenomenon is becoming increasingly common in South Korean nursing schools [12, 13].

Despite the growing awareness of the seriousness of student incivility, previous studies on incivility in the nursing education context in South Korea have focused only on incivility that students experience [12, 14] and uncivil behavior among nursing students [15]. Meanwhile, many nursing scholars have emphasized the need for research to reveal lived experiences and suggest alternative methods to manage student incivility through the eyes of faculty members in the nursing education context [2, 5, 16]. Therefore, this study aims to explore nursing faculty members’ experiences with student incivility in South Korea in terms of uncivil behavior by students, their responses to student incivility, consequences of student incivility, and alternative methods to deal with student incivility to solve associated problems. The current study may offer a knowledge base for academic incivility in nursing education to provide direction for evidence-based strategies aimed at developing civil nursing education environments.

## Methods

### Design

We used an exploratory qualitative design to describe the nursing faculty members’ experiences with undergraduate student incivility in the education context. Our preunderstandings are drawn from experiences with students in clinical and classroom teaching; however, we have made efforts to exclude them throughout the research process. We defined incivility as any speech or action that is disrespectful, rude, or shows disregard for others [17].

### Participants

Individuals who had been teaching in college for at least 1 year and had experienced student incivility were eligible for the study. First, we contacted potential participants by e-mail or telephone, explained the purpose of the study, and asked them to participate in our study. Using a purposive sampling method, we conducted individual interviews with the faculty who accepted our invitation to participate in the study. Data saturation, in which no new data or codes were extracted, was achieved after finishing the interview with fourteen faculty members. All of the participants were female, full-time faculty members who served as part-time faculty prior to becoming full-time. The average age was 46.3 (range: 39 to 62), and the average number of years in nursing education was 10.7 (range: two to 36). The participants varied from junior to senior faculty members, who were teaching various subjects, such as adult nursing, women’s health nursing, mental health nursing, and community health nursing. The participants were also teaching clinical practicum.

### Data collection

In-depth interviews were conducted by three researchers who were not affiliated with the participants’ schools and were experts in qualitative research. Field notes were used to record the participants’ nonverbal communications, such as facial expressions and intonation. The interviews were conducted in quiet rooms at the participants’ schools or places convenient for them and were audio-recorded after obtaining the participants’ permission. Every participant was interviewed once or twice (depending on data saturation), and each interview lasted 40 to 50 min.

To ensure that the interview questions were the same for all participants, we used an interview guide (Table 1). Our main questions were (a) “What kinds of uncivil behavior from students have you experienced in the class, in the clinical practicum setting, or outside the class?”; (b) “How do you respond when you encounter uncivil behavior?”; (c) “What do you think are the consequences of student incivility in nursing education?”; and (d) “What do you think are the alternatives in addressing student incivility?” The data were collected during January 2016.

**Table 1:** Interview guide

Divisions	Interview questions or directions
Introductory	● Incivility can be defined as any speech or action that is disrespectful, rude, or shows disregard for others. ● When you think the word ‘student incivility’, how do you feel about?
Main (key) questions	● What kind of uncivil behaviors from students have you experienced in the class, in the clinical practicum setting, or outside the class? ● How do you respond when you encounter those uncivil behaviors? ● What do you think are the consequences of student incivility in nursing education? ● What do you think are the alternatives in addressing student incivility?
For probing	● Please tell me more about that. ● Please talk about what you have experienced.
Closing question	● Is there anything you would like to add?

### Data analysis

We transcribed the interviewees’ narratives verbatim and then employed a qualitative content analysis method to analyze them [18, 19]. Every transcript was read multiple times so that we could grasp and familiarize ourselves with the participants’ experiences and obtain a sense of the whole data set. Furthermore, we reviewed the memos recorded during the analytic process. Data deemed relevant to the participants’ experiences with student incivility were identified and coded. The coded data were grouped into subthemes by comparing the meanings of the codes in relation to all raw data. Afterward, the subthemes were also grouped into thematic categories based on similarities and differences in their meanings. Ultimately, 6 themes and 15 subthemes were derived from 38 codes.

### Trustworthiness

To ensure the trustworthiness of the study’s results, we followed the criteria (credibility, dependability, transferability, and confirmability) by Lincoln and Guba [20]. To establish credibility, we constructed thorough and vivid descriptions of the participants’ experiences. We also conducted member checking and peer checking. For peer checking, we asked two faculty members who had experiences with student incivility to confirm the findings of the analysis. For member checking, we also asked two qualitative researchers to confirm the codes, subthemes, and themes. For dependability, the three researchers who conducted individual interviews with participants used the same interview guide. We also recorded memos that showed the decisions made during the analytical process. For transferability, we included participants from various age groups who were teaching various subjects and clinical practicum. Additionally, we provided rich descriptions of the findings with appropriate quotations from the participants. For confirmability, we tried to close the gap between the results from the analysis and the participants’ actual experiences, and we developed an audit trial for the interview data, analytical process, field notes, and memos used.

### Ethical considerations

The study was approved by the Institutional Review Board (KYUH 2015-01-014) of the university. All the participants voluntarily agreed and provided written informed consent to participate in the study, and we obtained their permission to audio record the interviews. All participants were informed that the study results would be reported anonymously and that they could withdraw from the study at any time, for any reason, without penalty. To ensure confidentiality, all interview data, related notes, and recorded files were stored on the hard drive of a password-protected computer accessible only to the authors.

## Results

The participants’ experiences with student incivility toward faculty members were described in the following six themes (Table 2): (1) nursing student indifference to teachers’ expectations, (2) ignoring behavioral standards in school life, (3) differing responses to uncivil student behavior, (4) self-reflection as a nursing educator, (5) negative impact on faculty in a nursing education context, and (6) awareness of civility in a nursing education context.

**Table 2:** Teachers’ experiences with student incivility: themes, and subthemes

Themes	Subthemes
Nursing student indifference to teachers’ expectations	● Disrespect toward faculty in a nursing education context ● Unfaithful in academic learning
Ignoring behavioral standards in school life	● No concern over what is right and wrong in one’s behaviors ● No respect for personal boundaries with nursing faculty
Differing responses to uncivil student behavior	● Publicly pointing out a nursing student’s uncivil behavior
	● Abdicating the role of guides to nursing students who are uncivil
Self-reflection as a nursing educator	● Concern over age differences with nursing students ● Introspection about oneself and one’s teaching
Negative impact on faculty in a nursing education context	● Threats to self-esteem as a nursing educator
	● Disruption of the teacher-student relationship in a nursing education context
	● Becoming desensitized to the culture fostered by the students’ uncivil behaviors
	● Loss of passion for teaching nursing student
Awareness of civility in a nursing education context	● Helping nursing students recognize uncivil behaviors
	● Efforts to foster a climate of civility in nursing school
	● Teaching nursing student about humanism

### Nursing student indifference to teachers’ expectations

#### Disrespect toward faculty in a nursing education context

Common examples of student incivility reported by the participants include not greeting teachers, showing up unannounced, breaking appointments, appearing bored during lectures, expressing defiance, and insincere responses to teachers’ questions. The participants stated that they felt disrespected when students behaved in these ways. Participant D stated:

*One day when I went on a business trip, I got a call from a student. The student said, “I dropped in at your office today, and you were not there. I need a reference [for a job application] that should be submitted to the hospital by tomorrow.” Even if I had gone to school then, I would not have arrived until 10 pm, and it would not have been possible to meet the student at 10 o’clock at night. Students do not come on time when they need something like a reference. They always come when the deadline has passed and, even then, without informing me of their visit beforehand. I already told senior students to prepare in advance … but students do not care about my advice. When they need something, they just show up … It seems to me that students do not regard the professor as deserving respect but as someone who meets their needs.*

#### Unfaithful in academic learning

The participants told us that students did not seem to be faithful in academic learning and to take nursing education seriously. Students were late to class; during class, they ate, applied make-up, or talked and played on their mobile phones; and they left whenever they pleased. During the clinical practicum, students did not seem genuinely engaged. For example, students did not wear the uniform required for the practicum, came to the hospital for the clinical practicum wearing the uniform under a coat, and complained about the length of practice time, even regarding surgery in the operating room. Additionally, students did not seem to be aware of patients’ rights to privacy and confidentiality. Students seemed to consider patient case studies in the clinical practicum as something they could use to obtain a good grade and not as involving individuals to be protected and respected.

### Ignoring behavioral standards in school life

#### No concern over what is right and wrong in one’s behaviors

The participants stated that the students showed little accountability and concern over what is right or wrong in their behavior as learners. Students would ask for grade changes and complain about test questions but not reflect on or take responsibility for the inappropriateness of their own behavior. In addition, students did not take their teachers’ reprimands about inappropriate behaviors seriously. Participant L commented:

*When students send me emails to ask for their grades, they only state their school identification numbers and names without informing me of their contact information. Sometimes students send texts to me very early in the morning or in the middle of the night. Students state what they want the teacher to do for them without any apology or politeness, and they mention their personal business without considering other people’s [their teachers’] situations.*

#### No respect for personal boundaries with nursing faculty

The participants described students as not respecting the personal boundaries of faculty members and requesting unreasonable favors from them. For example, students made demands for different test times, changes to due dates for papers, and more time to upload lecture materials. Students did not seem to differentiate between behaviors that are appropriate with friends and those appropriate with teachers, and they treated professors like fellow students; they even made personal and negative comments on anonymous course evaluations of the faculty. Participant A said:

*I was preparing lecture materials for next week’s class, and it was 9 p.m. when I uploaded the class materials. The next day, I went to school and informed students, via e-bulletin board, that I had posted class material. Then, I got a text from a student stating “I want you to do it before six p.m. before the library closes.” I got that text at 10 a.m. in my office. It made me uncomfortable because the students were thinking too much about their own convenience.*

### Differing responses to uncivil student behavior

#### Publicly pointing out a nursing student’s uncivil behavior

The participants said that when they dealt with student incivility, they repeatedly tried to correct the behavior, even if they were offended by the students’ comments. Participant D described:

*When I pointed out students’ incivility, I was prepared to be reviled by them. When I entered the classroom, if the students were talking, I hit my desk to get the students to concentrate or I nag them to get their attention. I had to nag a lot to get them to pay attention during class.*

#### Abdicating the role of guides to nursing students who are uncivil

Some participants stated that they tended to avoid confronting students about their uncivil behavior by not responding to such behavior. Participant E reported the following incidents:

*One day, students were whispering with others during class time, and it interrupted my focus, so I pointed it out. The students seemed to be embarrassed by this. After that, I began to pass it over without any response.*

Another participant (F) said:

*I don’t point out students’ incivility during class time. I just move on. Actually, I think I should point it out and correct them, but I am ambivalent about doing that. On the one hand, I understand it is hard for students to sit and concentrate in class all the time.*

### Self-reflection as a nursing educator

#### Concern over age differences with nursing students

The participants told us that they felt students and teachers have different perceptions of what constitutes acceptable behavior due to differences in norms between the generations. In trying to identify and resolve possible misunderstandings with students, the participants often attributed the cause to their age differences. A typical participant comment was “Is it common for all students to act like that these days? If I point out student behavior, students will think of it as an adult nagging. If I were to point it out, students would perceive me as an old-fashioned person.”

#### Introspection about oneself and one’s teaching

The participants reflected on their teaching role and described their responses to students’ incivility during class time. First, they examined their own behavior before pointing out uncivil behavior to the students. They also tended to address the problems in the context of immediate teacher-student interaction. The participants stated that they were afraid that their reprimands in response to incivility would be interpreted as an abuse of power. Participants G stated:

*I take time to think back on whether there was a problem in my teaching method or whether there was any misunderstanding with the student indicating there was something wrong with me.*

### Negative impact on faculty in a nursing education context

#### Threats to self-esteem as a nursing educator

The participants indicated that they felt their self-esteem as teachers was threatened because students seemed to ignore them. They stated that they were uneasy and felt embarrassed about confronting student incivility. Participant E stated:

*I feel ashamed of myself as a professor. I think as a teacher, my role is to help the students become professional nurses, and when I encounter student incivility, I get discouraged.*

#### Disruption of the teacher-student relationship in a nursing education context

The participants expressed concern that persistent student incivility may disrupt teacher–student relationships and widen the psychological distance between them. The participants explained that their ability to establish sincere relationships with the students had weakened and expressed concern over the possibility of losing compassion or affection for the students. Participant A stated:

*Because students’ uncivil behaviors negatively influence the educational context, relationships between students and their professors are broken; the closeness between them is also broken.*

#### Becoming desensitized to the culture fostered by the students’ uncivil behaviors

Some participants reported that they became accustomed to incivility and its consequences when student incivility persisted, and they accepted that they were teaching in an environment not conducive to a positive learning experience. The participants felt they were becoming desensitized to a culture of incivility. Participant B stated:

*Because I knew that students hated receiving negative or critical feedback about their behavior—even uncivil behavior—from teachers, I gradually stopped caring about the students’ inappropriate behavior like eating, texting, and putting make-up on during class. In the meantime, I was becoming desensitized to the students’ negative behaviors.*

#### Loss of passion for teaching nursing student

The participants told us that, due to persistent incivility from students, they gradually lost their passion for teaching nursing students. They stated that they recognized that there is no need to work hard, and their interest in being a good teacher gradually cooled. Participant F stated:

*I was offended to see students bowing down to their seniors while not greeting me, the professor who taught them. And, when I see that students react differently depending on the professor’s inclination and that students do not greet the so-called good professor, I felt my desire to be a good teacher diminished. He also felt his affection for the students waning.*

### Awareness of civility in a nursing education context

#### Helping nursing students recognize uncivil behaviors

To create a culture of civility at school, the participants wanted their students to first recognize what constitutes uncivil behavior. In other words, they thought that professors should help students define clear boundaries between right and wrong behavior in the educational environment. They also thought that teachers should give feedback about these behaviors and not avoid or ignore them. Participant A explained her approach as follows:

*I think I should point out the uncivil behavior even though it makes me very tired. I should confront students’ incivility even though I would be more comfortable avoiding it.*

Another participant (C) stated:

*Before, I did not point it out whenever I witnessed it. Now, I think if we continue to give them feedback about their behavior, they will know which behaviors are unacceptable and then they can gradually fix them.*

#### Efforts to foster a climate of civility in nursing school

The participants said there was a need to foster a climate of civility in school. They indicated that because civil behavior is a habit and an everyday activity, teachers need to repeatedly reinforce the need for good manners. They felt that it is necessary for students to be comfortable in a civil atmosphere that emphasizes courtesy. They further pointed out that etiquette and civil behavior should be natural in college, and to achieve that, students need to strongly value the wellbeing of others. Participant A told us about her approach: “I will repeatedly teach etiquette and civility to the students. I believe the students can be accustomed to civility if they learn it during the four years.” Another participant (B) stated, “Students went to college from high school without developing values about right and wrong, so college needs to provide an opportunity to form these values.”

#### Teaching nursing student about humanism

The participants indicated that to create a culture of civility at school, the institution should provide formal courses in humanism rather than just discussing or warning the students. They indicated that teaching humanism in school will help students instill a spirit of respect for human beings and move away from results-oriented thinking. Participant C stated:

*I heard that hospital care team members welcome students who learned humanism at school. If graduate students started working in hospitals without learning humanism first, they would be immature, impatient, and inconsiderate. Personality is formed and developed throughout life from kindergarten to elementary school, middle school, high school, and then college, even until one is old. I think that college students should learn humanism even though they are adults.*

## Discussion

Academic incivility by students is a challenging issue in nursing education around Western countries [6, 21, 22] as well as oriental countries [1, 4, 23]. Incivility is not just about a particular behavior; it has implications in various contexts, and its impact can be vast and serious. It has an adverse effect on individual faculty members and negatively influences the relationships between faculty and students, which further affects the education field [2, 5, 16]. Nursing students develop their practical competence through college education, where they are simultaneously experiencing a socialization process, learning professional speech, behavior, and culture [21]. Consequently, issues of student incivility in the educational context can spread from academia to the clinical field [16]. If students’ incivility is not adequately addressed, it can be carried forward to their professional behavior when they become nurses and enter clinical practice, causing serious issues and leading to poor patient care, patient dissatisfaction with the care provided, and interpersonal problems in the working ward [2].

The participants reported student incivility during and outside of class time. Some of the common uncivil behavior students exhibited included not greeting teachers, showing up for meetings unannounced, and having defiant attitudes, which faculty perceive as disrespectful. Similar uncivil behavior was also reported in previous research [21]; however, there were cultural differences across countries. A study of a nursing faculty in Iran reported disrespectful behaviors such as not standing up when a teacher entered a room and walking in front of a teacher [4]. In Iranian culture, this kind of behavior is considered socially deviant. However, in other cultures, such as South Korea, this kind of behavior is not a primary concern. At the other extreme, Luparell [6] reported that nursing faculty in the United States felt that the physical safety and wellbeing of their families and themselves were threatened by student incivility. Although our participants did not mention such serious incidents, uncivil behavior in educational environments should be addressed regardless of severity [6, 21]. It has been reported that college students prefer professors to manage classroom incivility because it also infringes on student learning [24]. Therefore, we need to manage students’ uncivil behavior to create a positive learning environment.

Our participants indicated that their students were uncommitted to academic learning and did not take nursing education seriously. One case of uncivil behavior encountered occurred when students came to the hospital for a clinical practicum wearing their uniform under a coat. This was especially uncivil and unprofessional, as it can lead to infection in vulnerable patients. In prioritizing personal convenience and efficiency, millennial generation students commit academic incivility that will carry over to the clinical and professional setting when they graduate and become nurses [5, 10]. Clark and Springer [21] previously noted another characteristic of students, that is, a sense of entitlement for job acquisition, which also contributed to student incivility in nursing education. Attitudes of entitlement among students can be one of the main factors increasing uncivil conduct among nursing students [23]. Clark and Springer [2] also noted that student incivility can lead to compromised patient care and safety when students graduate and work in hospitals. Therefore, efforts to educate students about academic civility and engaging in learning faithfully are essential.

Some participants stated that their students showed no concern regarding the right and wrong in one’s behavior, specifically, by ignoring school code of conduct standards and lacking accountability for their actions. The nursing profession has ethical standards, and as undergraduates, students should abide by them to ensure professional behavior after graduation [21]. Thus, holding students accountable and requiring that they establish the values of right and wrong will help them grow into professional nurses [3]. The values for right and wrong are not established within a short period of time. They are slowly formed and rooted throughout adulthood. Many studies [23, 25, 26] have recommended strategies to help students learn the values of right and wrong behavior and ethical standards of the nursing profession. Ward and Yates [25] suggested using the freshman orientation or course syllabi to explain policies regarding their code of conduct and civility policies. Another suggestion was for nursing faculty members to act as role models by exercising good listening, interpersonal skills, and respectful attitudes because students can learn civil behavior by observing faculty role models [2, 27]. Additionally, a strategy of student-generated rules about behaviors in the class and clinical practicum has also been suggested [26]. As students become more senior, the curriculum tends to focus more on subjects related to clinical practical competency. Therefore, initial humanities subjects that provide the opportunity to think about dignity and human values should be added to the curriculum.

When confronted with student incivility, our participants tended to either respond reactively or ignore the situation. Yassour-Borochowitz and Desivillia [7] proposed that the tendency of faculty to ignore incivility was the main reason for persisting uncivil behaviors among students; furthermore, faculty members’ inconsistent attitudes - react or ignore - toward student behavior draw unclear boundaries between acceptable and unacceptable behavior. Rad and Moonaghi [28] also reported that faculty overlook incivility because they lack relevant management skills and/or strategies. Indeed, students may require immediate responses to incivility during class [21]. McNaughton-Cassill [29] emphasized the need for faculty to enhance their capacity to manage student incivility because the perception of the faculty’s competence among students influences how students respond in class. Students often regard silence as ignorance or tolerance of their uncivil behavior [30]; therefore, requiring faculty members to respond more actively and confidently to student incivility will help deter this behavior.

The participants who reported self-reflection as nursing educators indicated their concern over the generation gap between faculty members and students. These findings support a study by Rad and Moonaghi [28], who suggested that faculty should consider the differing views between students and faculty. In particular, Ziefle [24] noted that values varied due to the generation difference between faculty and student. Generation X students value independence and work-life balance, while the Baby Boomer generation, most of the faculty, values loyalty and the centrality of work. As our world changes rapidly, interests, standards, and values are evolving as well. We are required to prepare for the trend that the nursing education field becomes more individualistic, market-oriented, and consumeristic [7]. Consequently, faculty members need time to review the effectiveness of their current teaching methods and to implement new methods that provide high-quality education for students in this new educational milieu [30].

To close the gap between faculty members and students, Rad and Moonaghi [28] suggest that faculty members forge interactive rather than authoritative relationships with their students. This will encourage open discussion of diverse opinions, viewpoints, and beliefs. Such open dialogs among students, faculty, and administrators will encourage the exchange of new ideas and concerns [31, 32]. Faculty members need to acknowledge differing opinions without being defensive [29]. Blogging is a particularly innovative teaching tool that can motivate technologically focused millennial students who are familiar with communicating their thoughts, ideas, and opinions through social networking systems [33]. While many faculty members may be unfamiliar with integrating social networking into the teaching curriculum, this new teaching method can better meet the needs of students while also creating an interactive relationship that limits incivility. Through self-reflection, faculty members can improve themselves behaviorally, making them better role models to students; consequently, it may help students establish appropriate standards of behavior [28].

Regarding the negative impact of student incivility on faculty members, our participants noted that incivility negatively affected their self-esteem as nursing educators and their desire to be good teachers, which was an internationally common response in previous studies [4, 6]. Disruption of the teacher-student relationship was also another negative consequence identified in previous studies [4, 5, 34]. Like human behavior, incivility between faculty and students is a reciprocal and dynamic process [33]. Student incivility not only negatively affects faculty members and the relationship between faculty and students but also disrupts other students in class, further interfering with the learning process. As such, it is essential that faculty members address rather than ignore uncivil behavior.

Our findings on the consequences of incivility were somewhat different from those of previous studies conducted in Western countries, which found that student incivility may tarnish teachers’ reputations [5], cause harm to the health and wellbeing of faculty members [6], and lead to faculty shortages [35]. Our participants did not report such serious consequences, which likely relates to cultural differences. In South Korea, Confucianism is rooted in tradition and is characterized by a culture of honoring the elderly [36]. In addition, respect and etiquette in social-rank relations according to age are still important [37]. Such cultural characteristics are still permeated to the students, although the new generation of Korean students is changing. Further research should explore experiential differences across cultures to help identify factors that contribute to extreme student incivility.

Despite cultural differences in student incivility across countries, our participants agreed that students need to be educated in the matter to foster a culture of civility in a nursing education context. Specifically, students should be able to define clear behavioral boundaries and to receive feedback on their behavior. Higher education goes beyond simply learning knowledge and skills; thus, behavioral standards for both schooling and professional careers must be part of the nursing curriculum [4]. Simultaneously, schools should guide students in prioritizing the wellbeing of others; they should emphasize both emotional intelligence and intellectual achievement.

Addressing incivility in nursing education will help enhance the quality of the learning process worldwide [29]. Faculty members are in a key position to create a civil culture in education. They can be role models for students who are in the process of transitioning to professional nurses [30]. The present study has significance in that it delineated the uncivil behaviors experienced by the faculty and the negative effects caused by student incivility in the educational context. Furthermore, our study suggests alternatives to managing student incivility and ways to foster a positive educational environment through the eyes of the faculty. The findings suggest a more evidence-based direction to foster a safe and comfortable learning environment for students and faculty members.

## Limitations

One limitation of this study was its lack of male participants. Females dominate nursing education in South Korea, although the proportion of male teachers is increasing. Future studies should include a larger proportion of male faculty members and examine gender differences in experiences with student incivility. In this study, the faculty’s experiences with student incivility did not show differences across student gender. However, we can consider the gender issues in some aspects, in terms of the difference in uncivil behavior according to the student’s gender and in the faculty member’s experiences of student incivility by a student’s gender. There is no research that has reported a difference in the findings by gender. However, studies have reported a difference in the student incivility experience according to the gender of the student [15, 17]. Therefore, future studies should identify such gender differences in various ways. Another limitation is the potential for researcher bias on student incivility because researchers are part of nursing faculty. However, as mentioned earlier, we have taken steps to ensure rigor in research and reduce bias. In addition, all the participants were full-time faculty members. Rawlins [32] suggested that incivility between faculty members and students may vary among part-time versus full-time faculty members. Therefore, future studies need to examine the difference between student incivility according to the faculty member’s status.

## Implications

This study has several academic and educational implications. First, this study will help understand the faculty experiences of student incivility, which reflect the characteristics of South Korean culture in a nursing education context. Second, it will also contribute to realizing the effect of generational differences between faculty members and students on the learning process. Third, it provides direction for fostering a civil climate and developing strategies to address student incivility in nursing education.

## Conclusions

The present study showed the uncivil behavior experienced by the faculty and the resulting negative effects in the educational context. Additionally, it suggested alternatives to manage student incivility and foster a positive educational environment from the faculty members’ perspective. We found that student incivility negatively influenced faculty members on a personal level and degraded the teacher–student relationship. The participants’ experiences showed that student incivility exerts a strong influence on the educational process. They highlighted a need to manage incivility and outlined ways to foster a culture of civility in nursing education. The findings suggest an evidence-based direction to foster a safe and comfortable learning environment for students and faculty members. Future studies are thus needed to identify determinants of incivility, assess factors that foster a culture of civility, and devise methods for developing civil education for nursing students.

## Data Availability

The data that support the findings of the current study are fully suggested within the study.

## References

[1] Eka NGA, Chambers D, Narayanasamy A (2016). Perceived uncivil behaviour in Indonesian nursing education. Nurse Educ Pract.

[2] Clark CM, Springer PJ (2010). Academic nurse leaders’ role in fostering a culture of civility in nursing education. J Nurs Educ.

[3] Ibrahim SAE, Qalawa SA (2016). Factors affecting nursing students’ incivility: as perceived by students and faculty staff. Nurse Educ Today.

[4] Masoumpoor A, Borhani F, Abbaszadeh A, Rassouli M (2017). Nursing instructors’ perception of students’ uncivil behaviors: a qualitative study. Nurs Ethics.

[5] Sprunk EA, LaSala KB, Wilson VL (2014). Student incivility: nursing faculty lived experience. J Nurs Educ Pract.

[6] Luparell S (2004). Faculty encounters with uncivil nursing students: an overview. J Prof Nurs.

[7] Yassour-Borochowitz D, Desivillia H (2016). Incivility between students and faculty in an Israeli college: a description of the phenomenon. Int J Teaching Learn Higher Educ.

[8] Clark CM, Olender L, Cardoni C, Kenski D (2011). Fostering civility in nursing education and practice: nurse leader perspectives. J Nurs Adm.

[9] You M, Shim H (2013). A study on the cultural characteristics of Korean society: discovering its categories using the cultural consensus model. Kor J Psychol: Cul Soc.

[10] Kim E (2012). Characteristics of the current student generation and considerations for medical education. Kor Med Educ Rev.

[11] Kim J (2002). The education that the new generation wants receive and the education that the older generation wants to give. Educ Dev.

[12] Hyun MS, De Gagne J, Park J, Kang HS (2018). Incivility experiences of nursing students in South Korea. Nurs Ethics.

[13] Park EJ, Park CS (2020). Research trends of misbehavior involving nursing students: a text network analysis. J Educ Cult.

[14] Kim SA, Lee SY, Hong E (2019). The effects of a communication program on incivility, critical thinking, and clinical practice stress experienced by nursing students. J Kor Acad Soc Nurs Educ.

[15] Park JH, Jang EY, Lee SM (2015). Factors affecting incivility experience among nursing students. J Med Stat Inform.

[16] Milesky JL, Baptiste D, Foronda C, Dupler AE, Belcher AE (2015). Promoting a culture of civility in nursing education and practice. J Nurs Educ Pract.

[17] Clark CM (2008). Student voices on faculty incivility in nursing education: a conceptual model. Nurs Educ Perspect.

[18] Downe-Wamboldt B (1992). Content analysis: method, application, and issues. Health Care Women Int.

[19] Granehein UH, Lundman B (2004). Qualitative content analysis in nursing research: concepts, procedures and measures to achieve trustworthiness. Nurse Educ Today.

[20] Lincoln YS, Guba EG (1985). Naturalistic inquiry.

[21] Clark CM, Springer PJ (2007). Thoughts on incivility: student and faculty perceptions of uncivil behavior in nursing education. Nurs Educ Perspect.

[22] Schaeffer A (2013). The effects of incivility on nursing education. Open J Nurs.

[23] Natarajan J, Muliira JK, van der Coljj J (2017). Incidence and perception of nursing stucents’ academic incivility in Oman. BMC Nurs.

[24] Ziefle K (2018). Incivility in nursing education: generational differences. Teach Learn Nurs.

[25] Ward C, Yates D (2014). Civility in the university classroom: an opportunity for faculty to set expectation. Contemp Issues Educ Res.

[26] Duplaga E, Astani M (2010). An exploratory study of student perceptions of which classroom policies are fairest. Decis Sci J Innov Educ.

[27] Knepp KF (2012). Understanding student and faculty incivility in higher education. J Effect Teach.

[28] Rad M, Moonaghi HK (2016). Strategies for managing nursing students’ incivility as experienced by nursing educators: a qualitative study. J Caring Sci.

[29] McNaughton-Cassill ME (2013). Is it incivility or mental illness? Understanding and coping with disruptive student behavior in the college classroom. J Effect Teach.

[30] Clark CM (2009). Faculty field guide for promoting student civility on the classroom. Nurse Educ.

[31] Hoffman EM (2014). Faculty and student relationships: context matters. Coll Teach.

[32] Woodworth JA (2016). Promotion of nursing student civility in nursing education: a concept analysis. Nurs Forum.

[33] Jones K, Garrity MK, VanderZwan KJ, Epstein I, Burla de la Rocha A (2016). To blog or not blog: what do nursing faculty think?. J Nurs Educ.

[34] Ingraham KC, Davidson SJ, Yonge O (2018). Student-faculty relationships and its impact on academic outcomes. Nurse Educ Today.

[35] Rawlins L (2017). Faculty and student incivility in undergraduate nursing education: an integrative review. J Nurs Educ.

[36] Yang J (2020). Korean university students’ perception on intergenerational communication: focusing on cultural factors. J KoCon A.

[37] Shim K, Inumiya Y, Yoon S, Suh S, Zhang Y, Han S (2012). A study on the development of Confucian values scale. Kor J Clin Psychol: General.

